# Mutation of mitochondria genome: trigger of somatic cell transforming to cancer cell

**DOI:** 10.1186/1755-7682-3-4

**Published:** 2010-02-05

**Authors:** Du Jianping

**Affiliations:** 1 Department of Oncology, Anhui Provincial Hospital, 9 Lujiang Road, Hefei 230001, PR China

## Abstract

Nearly 80 years ago, scientist Otto Warburg originated a hypothesis that the cause of cancer is primarily a defect in energy metabolism. Following studies showed that mitochondria impact carcinogenesis to remodel somatic cells to cancer cells through modifying the genome, through maintenance the tumorigenic phenotype, and through apoptosis. And the Endosymbiotic Theory explains the origin of mitochondria and eukaryotes, on the other hands, the mitochondria also can fall back. Compared to chromosome genomes, the mitochondria genomes were not restricted by introns so they were mutated(fall back) easy. The result is that mitochondria lose function and internal environment of somatic cell become acid and evoked chromosome genomes to mutate, in the end somatic cells become cancer cells. It is the trigger of somatic cell transforming to cancer cell that mitochondria genome happen mutation and lose function.

## Cancer energy metabolism and mitochondria

Nearly 80 years ago, scientist Otto Warburg observed that cancer cells perform energy metabolism in a way that is different from normal adult cells, so he originated a hypothesis that the cause of cancer is primarily a defect in energy metabolism [1]. With the mutational theory of carcinogenesis(including oncogenes and tumor suppressor genes) discovering, Warburg' theory has expressed shortage, but mitochondria engageing process of carcinogenesis become to highlight, Cavalli suggest that mitochondria impact carcinogenesis through mitochondrial DNA acting as transposable elements and modifying the genome, through mitochondrial maintenance of the tumorigenic phenotype, and through the role of mitochondria in apoptosis [2].

## Cause of modification in glycolysis

Since the days of Warburg, studies have continued to show alterations of functional energy metabolism in cancer cells. The cancer cells look like to be function losed cells and only energy consuming cells, they survive just for surviving and proliferating. Among these differences are increased rate of glycolysis [3], and shifts in LDH isozyme patterns [4], and ceased production of functional proteins [5]. The high glycolysic rate maintained by proliferating cells and tumor cells may possibly be due to altered expression of enzymes [6]. Glycolysis may be stimulated by availability of ADP(a rate-limiting step), and it has been suggested that either inefficient ATPase activity(the Na+/K+ atpAse in Ehrlich ascites tumor cells) leading to increased ATP hydrolysis [7] or increased ATPase activity may contributed to the increased ADP levels [8]. In effect, the cancer cell almost stop using the citric acid cycle to get energy.

## Traditional theory on mutution of mitochondrial genome

In terms of the mitochondrial genome, there is some evidence for the transfer of mitochondrial DNA into the nuclear genome, even in the human cell [9]. There ie also abundant evidence for alteration of transcription rates for the mitochondrial genome in cancer cells [10]. Mitochondrial DNA may also be more susceptible to mutution [11]. This may be due either to its supercoiler struture [12] or the paucity of repair mechanism for Mitochondrial DNA [13]. Another contributing factor may be the relatively constant exposure of Mitochondrial DNA to free radicals produced by the respiratory chain. Finally, study of hamster kidney tumor cells indicated that there is a reduction in Mitochondrial DNA in those cells, which may have implications for functional energy metabolism in those cells [14]. Above all, there may be another reason why mitochondrial genome easly mutate, that is from Endosymbiotic Theory.

## Endosymbiotic Theory

In the 1960's, Lynn Margulis found that their many similarities between prokaryotic cells and the organelles of eukaryotic cells and established hypothesis officially in her 1981 book "Symbiosis in Cell Evolution". The theory maintains that ancestors of eukaryotic cells were "symbiotic consortiums" of prokaryote cells with at least one and possibly more species (endosymbionts) involved. In other words, perhaps oxygen breathing bacteria(mitochondria) invaded an anareobic amoebalike bacteria(original prokaryotic host cell), and each performed mutually benefiting functions. The bacteria would breathe for the anareobic amoebalike bacteria, and the amoebalike bacteria would navigate through new oxygen-rich waters in search of food. This way, each of the organisms would be benefiting from their symbiotic relationship as the waters and atmosphere of the Precambrian changed. The invaded bacteria were mitochondria [15], then random mutations in the form of deletions large and small seem to have eliminated nonessential genes from the endosymbiont-mitochondrial genome lineages [16]. But mitochondria genomes consist of a single circular molecule of DNA and have their own protein-synthesizing machinery.

## Reason mitochondrial genome mutation

From Endosymbiotic Theory, human normal cell and mitochondrial have a relative genomes and protein-synthesizing system [17], they work independently and live together harmony. And each human cell contains hundreds of mitochondrion and each mitochondrion contains several presumably identical circular DNA molecules, frequently, a tumor will contain identical mitochondrial mutations [18]. Why? Paula suggests that tumor-associated mtDNA mutations lead to increased production of reactive oxygen species (ROS), a by-product of mitochondrial oxidative phosphorylation, which can stimulate cell proliferation [19]. I hypothesis that contrary to chromosome genomes, mitochondrial genome lack introns, so missed introns result in mitochondrial genome vulnerable to be affected by malignant envirments, such as free radicals(reactive oxygen species), especially maybe the introns look like housekeeping genes? Without introns maintenance genomes stable, the mitochondrial DNA may be more susceptible to mutution with no introns. Above all, the mutation of mitochondrial genomes is easier than somatic cell genomes. In fact, mutations in mtDNA have been identified in various types of human cancer, such as Breast cancer, Ovary cancer, Colon cancer, et al [20].

## Questions need answer

It is certain that cancer cell contains mutation genes of chromosomes, and mutations in mtDNA also identified in cancer cell. For why these mutation happened, there are many hypotheses about the origin of cancer, from Otto Warburg's to Paula A.Kiberstis's, their focas is still on energy metabolism and relevant mechanisms. Still now, important questions to be addressed include (1) What is the exact role of mtDNA in cancer initiation and progression? (2) How do heteroplasmic mtDNA mutations arise and evolve to a homoplasmic state in cancer cells? (3) Is there a mechanistic link between mtDNA mutations, changes in respiration and ROS generation, and alterations in apoptosis? [21] The answers to these questions would undoubtedly advance our understanding of mitochondrial biology in cancer, but no man exactly knows. Tumorigenesis is a complex process involving the activation of oncognens, inactivation of tumor suppressors and deregulation. The findings that pro-apoptotic genes might act as tumor suppressors and anti-apoptotic genes can serve as oncogenes suggested that the balance between pro-apoptotic and anti-apoptotic modulates tumor growth [22]. What exactly happened firstly remain pondering when a somatic cell transform to cancer cell?

## My hypothesis

Mitochondrial DNA mutations have been observed in many types of human cancer [23]. **The reason why mtDNA is easily mutate when they copy that they lack introns**. And all mutation mtDNA are tumor-associated mtDNA. As Petron's experiment describes, the pathogenic mtDNA ATP6 T8993G mutation was introduced into the PC3 prostate cancer cell line through cybrid transfer and tested for tumor growth in nude mice. The resulting mutant (T8993G) cybrids were found to generate tumors that were 7 times larger than the wild-type (T8993T) cybrids [24]. The last experiment was by Park JS, they found that the cell line carrying the heteroplasmic ND5 mtDNA mutation showed significantly enhanced tumor growth. Their results indicate that the mtDNA mutations might play an important role in the early stage of cancer development, possibly through alteration of ROS generation and apoptosis [25]. After mutation of mitochondrial genome happened, I presume mitochondria stop normal oxygen respiration to hypo-oxygen respiration, the somatic cell atavism and internal environment of cell changes to be acid (lactic acid), Which John AP' hypothesis did image [26]. Then in the acidic environment the chromosome genomes transform to disorder then all kinds of malignant characters expressed. In conclusion, I hypothesis that mutation of mitochondria genome is the trigger of the normal cell transforming to cancer cells.

## Conclusions

Main conclusions of the Review: (1) Role of mitochondria is important in physiological function of cell. (2) Probability of mutation of mitochondria genome is higher than that of human normal cell. (3) Mutation of mitochondria genome induce alteration of inner environment in human normal cell. (4) Bad inner environment results mutation of chromosome genomes and total cell atavism. (5) Mutation of mitochondria genome: trigger of normal cell transforming to cancer cell.

## Author's information

Dr. Du Jianping is post-graduate student of The Third Military Medical University, a famous Medical University in PR. China, and was conferred PhD and MD. Now I am a physician of Oncology and work in province hospital. My department possesses 150 hospital beds and accepts 6000 man-time cancer patients. In long experience of study and clinic practice, I found that: (1) Role of mitochondria is important in physiological function of cell. (2) Probability of mutation of mitochondria genome is higher than that of human normal cell. (3) We maybe pay too much attention on mutation of chromosome genomes and ignore mutation of mitochondria now and then we make little progress on curing cancer recent years. (4) Endosymbiotic Theory was established and it enlarge our vision. (5) We maybe breakthrough predicament of cancer therapy. So I write this review to induce other people thoughts.
